# Supplementary material for: Bulk tissue cell type deconvolution with multi-subject single-cell expression reference
Source: Nat Commun. 2019 Jan 22;10:380. doi: 10.1038/s41467-018-08023-x (PMC6342984; doi:10.1038/s41467-018-08023-x)
Supplement: Supplementary file 1 — Supplementary Information [file 41467_2018_8023_MOESM1_ESM.pdf]

Bulk Tissue Cell Type Deconvolution with Multi-Subject Single-Cell Expression Reference

Wang et al.

Supplementary Information

Supplementary Notes

Supplementary Note 1: Construction of artificial bulk tissue RNA-seq data

We construct artificial bulk tissue RNA-seq data by summing up read counts across all cells from the same subject in the single-cell RNA-seq data. By way of construction, the cell type proportions of the artificial bulk data are equal to the observed cell type proportions in the single-cell data, and this allows us to compare estimated cell type proportions from various methods with the true proportions. **Supplementary Figure 1b** shows that the artificial bulk tissue RNA-seq data have similar gene expression as the real bulk RNA-seq data generated from the same subjects.

Supplementary Note 2: Impact of varying cell type proportions of artificial bulk data in deconvolution

Figure 2b in the main text shows the deconvolution results from MuSiC, NNLS, BSEQ-sc and CIBERSORT, and these results indicate that the alpha cell proportion is over-estimated by all methods except for MuSiC. To evaluate the impact of different cell type proportions in the bulk data on deconvolution estimates, we generated additional artificial bulk data to show that MuSiC can still reliably estimate cell type proportions even when the true cell type proportions in the bulk data are very different from the cell type proportions in the single-cell reference. In this newly constructed benchmark data, the single-cell reference stays the same while we construct the artificial bulk data from Xin et al.[1] by combining cells from 2 subjects with 75% alpha cells dropped. In this way, beta cells become the dominant cell type in the artificial bulk data, as expected for real bulk tissue. **Supplementary Figure 2c** shows that only MuSiC recovers the true cell type composition, revealing that beta cells are the major cell type in the artificial bulk data, whereas the other methods overestimate the alpha cell proportion, indicating that these methods are more likely to be influenced by the cell type proportions in the single-cell reference. This analysis also gives the likely explanation for why, in the Fadista et al.[2] data, all methods that rely on CIBERSORT marker genes grossly overestimate alpha cell proportion.

Supplementary Note 3: Impact of missing cell types in single-cell reference on deconvolution

One of the limitations of single-cell RNA-seq is cell loss during cell dissociation. This not only biases cell type proportions, but also leads to failure of detecting certain cell types, especially those rare cell types. In practice, the single-cell reference dataset might be incomplete, and not every cell type present in the bulk data is included in the single-cell reference. Since the deconvolution methods rely on observed cell types in the single-cell reference, it is important to evaluate whether cell type proportions can be reliably estimated when some cell types are missing in the single-cell reference.

We evaluate MuSiC, NNLS, BSEQ-sc and CIBERSORT with missing cell types (**Supplementary Figure 3, Supplementary Table 3**). The artificial bulk data consist of 6 cell types while the single-cell reference only consists of 5 cell types. The evaluation shows that when major cell types are missed, none of the methods can give accurate estimates. However,

the cell type proportions are estimated accurately by MuSiC when the missing cell type is not the dominant cell type in the bulk tissue.

Supplementary Note 4: Tolerance of bias in single-cell relative abundance on deconvolution

The protocol discrepancies between bulk and single-cell datasets may lead to estimation bias. To evaluate the degree of bias tolerance relative to the biological signal, we manually introduce noise to cross-subject average of the single-cell obtained relative abundance θ_g^k. Because of the constraint that $\sum_{g=1}^{G}\theta_g^k = 1$, we generate biased relative abundance by Dirichlet distribution, denoted by $\theta_g^{k'}$. Consider one cell type only. For simplicity, we drop the superscript k for cell type. We assume the relative abundances of G genes follow a Dirichlet distribution,

$$\left(\theta_1^{'}, \dots, \theta_G^{'}\right) \sim Dirichlet\big(t \times (\theta_1, \dots, \theta_G)\big), \tag{1}$$

where t is a scaling factor. The mean and variance of $\theta_g^{'}$ are θ_g and $\frac{\theta_g(1-\theta_g)}{t+1}$, respectively. By setting $t = 999, 1332, 1999$ and 3999, corresponding to $\frac{Var\left[\theta_g^{'}\right]}{E^2\left[\theta_g^{'}\right]} \approx \left(\theta_g(1+t)\right)^{-1} \geq 2, 1.5, 1$ and 0.5, we simulated 100 times the cross-subject average of relative abundance of 6 major cell types from Segerstolpe et al.[3] We deconvolved the artificial bulk data constructed by Xin et al.[1] (**Supplementary Figure 8c**) and MuSiC provides accurate cell type proportions even with biased relative abundance as input.

Supplementary Note 5: Robustness to single-cell dropout noise on deconvolution

Single-cell RNA-seq data are prone to gene dropout and the dropout rates can differ across datasets. To evaluate the robustness of MuSiC, NNLS, BSEQ-sc and CIBERSORT to dropout in single-cell data, we constructed artificial bulk data from the original scRNA-seq data and deconvolve it by single-cell data with additional dropout noise. Following Jia et al.[4], the dropout rate π_{jgc} is generated by

$$\pi_{jgc} = \frac{1}{1 + k\exp(k\ln X_{jgc})}, \tag{2}$$

where X_{jgc} is the observed read counts, k is the dropout rate parameters. The simulated read count $X_{jgc}^{'}$ follows distribution such that

$$P\left(X_{jgc}^{'} = X_{jgc}\right) = \pi_{jgc}, \quad P\left(X_{jgc}^{'} = 0\right) = 1 - \pi_{jgc}. \tag{3}$$

We evaluated four different dropout rates with $k = 1, 0.5, 0.2$ and 0.1 (**Supplementary Figure 8a-b**). In general, adding more dropout noise leads to lower MuSiC estimation accuracy. Compared with other methods, MuSiC consistently performs better in the presence of dropout noise.

Supplementary Note 6: Convergence of MuSiC with different starting points

MuSiC estimates cell type proportions by weighted non-negative least square (W-NNLS), which might be sensitive to the choice of starting values. To examine the convergence property of

MuSiC, we re-analyzed the data in **Figure 2b** to show convergence with different starting points. The artificial bulk data is constructed by Xin et al.[1] while the single-cell reference consists of 6 healthy subjects from Segerstolpe et al.[3] The cell type proportions of four cell types: alpha, beta, delta and gamma are estimated using MuSiC with different starting points are shown in **Supplementary Table 8**. W-NNLS converges to the same value regardless of the starting points (**Supplementary Figure 9**).

Supplementary Note 7: Complex models

More complex error models, such as the gamma may give better fit to data, but could be computationally more challenging to fit. Here our empirical results show that the Gaussian model already gives accurate estimates.

Supplementary Tables

Supplementary Table 1: Linear regression to examine the relationship between estimated cell type proportions (Segerstolpe et al.[3] as reference) and HbA1c levels. The fitted linear model is estimated cell type proportion ~ HbA1c + Age + BMI + Gender. Significant results (p value < 0.05) are highlighted.

Cell type		MuSiC			BSEQ-sc		
		Estimate	Std.Error	P value	Estimate	Std.Error	P value
**alpha**	(Intercept)	0.380382	0.207754	0.07125	1.351464	0.240052	3.26E-07
	HbA1c	-0.00203	0.027737	0.941834	-0.07377	0.032049	0.024249
	Age	-0.00097	0.001935	0.617836	0.002753	0.002236	0.222198
	BMI	-0.00167	0.007945	0.834127	-0.01711	0.00918	0.066449
	Gender	0.033135	0.042881	0.442221	-0.00638	0.049548	0.897869
**beta**	(Intercept)	0.877022	0.190276	1.71E-05	0.065847	0.046433	0.16047
	HbA1c	-0.0614	0.025403	0.01819	-0.00295	0.006199	0.635957
	Age	0.002639	0.001772	0.140873	0.000576	0.000433	0.187339
	BMI	-0.01362	0.007276	0.065293	-0.00162	0.001776	0.365258
	Gender	-0.07987	0.039274	0.04566	-0.00541	0.009584	0.574159
**gamma**	(Intercept)	0.008556	0.010504	0.417988	0.102201	0.024366	7.69E-05
	HbA1c	0.001047	0.001402	0.457785	-0.00278	0.003253	0.396334
	Age	9.21E-05	9.78E-05	0.349431	-0.00013	0.000227	0.570225
	BMI	-0.00057	0.000402	0.160731	-0.00207	0.000932	0.029738
	Gender	-0.00165	0.002168	0.450416	-0.00092	0.005029	0.855252
**delta**	(Intercept)	0.057678	0.010592	6.81E-07	0.015539	0.018715	0.409122
	HbA1c	-0.00106	0.001414	0.455427	0.002017	0.002499	0.422131
	Age	-0.00016	9.87E-05	0.12039	9.99E-05	0.000174	0.568316
	BMI	-0.0011	0.000405	0.008142	-0.00103	0.000716	0.154263
	Gender	0.000424	0.002186	0.846817	-0.00254	0.003863	0.512616
**acinar**	(Intercept)	-0.10619	0.131102	0.420638	-0.14553	0.052092	0.006672
	HbA1c	0.034967	0.017503	0.049519	0.019075	0.006955	0.007684
	Age	-0.00247	0.001221	0.046841	0.00066	0.000485	0.178153
	BMI	0.00662	0.005013	0.190883	0.002008	0.001992	0.316847
	Gender	0.05332	0.02706	0.052632	-0.02338	0.010752	0.032985

**ductal**	(Intercept)	-0.21745	0.141008	0.127428	-0.38952	0.232841	0.098686
	HbA1c	0.028474	0.018826	0.134781	0.058397	0.031086	0.064353
	Age	0.000863	0.001313	0.513005	-0.00396	0.002169	0.072066
	BMI	0.010341	0.005392	0.059097	0.019814	0.008904	0.029191
	Gender	-0.00536	0.029105	0.854406	0.038631	0.048059	0.424144

Supplementary Table 2: Linear regression to examine the relationship between estimated cell type proportions (Baron et al.[5] as reference) and HbA1c levels. The fitted linear model is estimated cell type proportion ~ HbA1c + Age + BMI + Gender. Significant results (p value < 0.05) are highlighted.

**Cell type**		**MuSiC**			**BSEQ-sc**		
		Estimate	Std.Error	P value	Estimate	Std.Error	P value
**alpha**	(Intercept)	1.000504	0.275906	0.000533	1.220529	0.187349	8.56E-09
	HbA1c	-0.0259	0.036835	0.48424	-0.06398	0.025012	0.012632
	Age	0.000234	0.00257	0.927855	0.001921	0.001745	0.274661
	BMI	-0.01137	0.010551	0.28475	-0.00681	0.007164	0.345275
	Gender	0.038364	0.056948	0.502676	-0.02104	0.038669	0.588048
**beta**	(Intercept)	0.315176	0.09427	0.001316	0.011001	0.016796	0.51455
	HbA1c	-0.02843	0.012586	0.026936	-3.70E-05	0.002242	0.986889
	Age	-0.00081	0.000878	0.361952	0.000142	0.000156	0.366396
	BMI	-0.00158	0.003605	0.661813	-0.00044	0.000642	0.498345
	Gender	-0.00927	0.019458	0.635249	-0.00079	0.003467	0.819685
**gamma**	(Intercept)	-0.0172	0.055935	0.759333	0.040372	0.011566	0.000827
	HbA1c	0.001227	0.007468	0.869925	7.31E-05	0.001544	0.962362
	Age	0.00085	0.000521	0.107295	-8.47E-05	0.000108	0.434521
	BMI	-0.00042	0.002139	0.843112	-0.0011	0.000442	0.015394
	Gender	-0.00998	0.011545	0.390355	-0.00048	0.002387	0.842519
**delta**	(Intercept)	0.043785	0.009622	2.12E-05	0.012347	0.016882	0.466922
	HbA1c	-0.00121	0.001285	0.349663	0.002763	0.002254	0.224153
	Age	-8.79E-05	8.96E-05	0.330262	5.00E-05	0.000157	0.751577
	BMI	-0.00093	0.000368	0.013618	-0.00101	0.000646	0.1226
	Gender	-0.00063	0.001986	0.753674	-0.00098	0.003484	0.780352
**acinar**	(Intercept)	0.002232	0.042169	0.957925	-0.23299	0.083467	0.006714
	HbA1c	0.013032	0.00563	0.023475	0.034902	0.011143	0.00251
	Age	-0.00062	0.000393	0.119068	-0.00015	0.000777	0.848086
	BMI	-0.0008	0.001613	0.621478	0.006564	0.003192	0.043362
	Gender	0.013342	0.008704	0.129687	-0.01866	0.017228	0.282488
**ductal**	(Intercept)	-0.3445	0.218745	0.119669	-0.05126	0.14	0.715354
	HbA1c	0.041276	0.029204	0.161852	0.026281	0.018691	0.164004
	Age	0.00043	0.002038	0.833485	-0.00188	0.001304	0.153951
	BMI	0.015109	0.008365	0.075051	0.002786	0.005354	0.604398
	Gender	-0.03183	0.04515	0.483036	0.04194	0.028896	0.151016

Supplementary Table 3: Evaluation of deconvolution methods when there are missing cell types in the single-cell reference. The missing cell type is shown in bold and the proportions in the bulk tissue data are shown in parentheses.

**alpha** (0.447)	RMSD	mAD	R	**beta** (0.137)	RMSD	mAD	R
MuSiC	0.13	0.09	0.72	MuSiC	0.04	0.03	0.98
NNLS	0.27	0.18	0.42	NNLS	0.12	0.08	0.86
BSEQ-sc	0.17	0.12	0.58	BSEQ-sc	0.12	0.08	0.87
CIBERSORT	0.12	0.09	0.77	CIBERSORT	0.09	0.06	0.91
**delta** (0.092)	RMSD	mAD	R	**gamma** (0.062)	RMSD	mAD	R
MuSiC	0.04	0.03	0.98	MuSiC	0.05	0.038	0.97
NNLS	0.12	0.08	0.82	NNLS	0.12	0.081	0.84
BSEQ-sc	0.12	0.08	0.85	BSEQ-sc	0.12	0.083	0.86
CIBERSORT	0.10	0.07	0.90	CIBERSORT	0.10	0.070	0.90
**acinar** (0.084)	RMSD	mAD	R	**ductal** (0.177)	RMSD	mAD	R
MuSiC	0.05	0.04	0.97	MuSiC	0.050	0.037	0.97
NNLS	0.11	0.07	0.85	NNLS	0.067	0.046	0.96
BSEQ-sc	0.14	0.10	0.79	BSEQ-sc	0.084	0.064	0.93
CIBERSORT	0.07	0.05	0.93	CIBERSORT	0.076	0.058	0.94

Supplementary Table 4: Summary of cell types of Park et al.[6] single-cell dataset. Park et al. sequenced 57,979 cells from healthy mouse kidneys and identified 16 cell types. As suggested in Park et al., we limited our consideration to the 13 confidently characterized cell types and eliminated CD-Trans and 2 novel cell types in our deconvolution analyses.

Cell Type	Abbr.	# Cell	% Cell	Cell Type	Abbr.	# Cell	% Cell
Endothelial	Endo	1,001	2.29	Fibroblast	Fib	549	1.26
Podocyte	Podo	78	0.18	Macrophage	Macro	228	0.52
Proximal tubule	PT	26,482	60.54	Neutrophil	Neutro	74	0.17
Loop of Henle	LOH	1,581	3.61	B lymphocyte	B lymph	235	0.54
Distal convoluted tubule	DCT	8,544	19.53	T lymphocyte	T lymph	1,308	2.99
Collecting duct principal cell	CD-PC	870	1.99	Natural killer cell	NK	313	0.72
Collecting duct intercalated cell	CD-IC	1729	3.95	Novel cell type 1	Novel 1	601	1.37
Collecting duct transitional cell	CD-Trans	110	0.25	Novel cell type 2	Novel 2	42	0.10

Supplementary Table 5: List of top 100 high weighted genes from the pancreatic islet analysis.

Rank	Segerstolpe	Xin	GSE50244	Rank	Segerstolpe	Xin	GSE50244
1	GCG	GCG	MALAT1	51	ITM2B	EIF4A2	RPS3A
2	TTR	MALAT1	EEF1A1	52	ENPP2	CTSD	RPL9
3	MALAT1	INS	TTR	53	ATP1A1	RBP4	SOD2
4	SERPINA1	TTR	FTH1	54	ANXA4	HNRNPH1	EIF4B
5	SPP1	FTL	GCG	55	HNRNPH1	BSG	HSPA8
6	B2M	PPP1CB	CPE	56	ALDOB	EEF2	PKM
7	FTH1	PCSK1N	GNAS	57	CD164	RPS3	SCG2
8	CHGA	CHGB	RPL4	58	HLA-A	PDK4	RPS24
9	PIGR	PSAP	APP	59	RIN2	SSR1	CD74
10	IAPP	CHGA	CTSD	60	ASAH1	SCD	SQSTM1
11	SST	EGR1	HSP90AA1	61	TMSB10	DNAJC3	TMBIM6
12	FTL	SRSF6	RPLP0	62	BSG	SAR1A	TXNRD1
13	CALM2	FTH1	RPL7A	63	CLDN4	GPX4	LCN2
14	CHGB	HSP90AB1	HSP90AB1	64	TMEM59	PLD3	RPL14
15	SERPINA3	SPINT2	HSP90B1	65	PPY	ATP6AP1	PDIA3
16	ACTG1	MAP1B	UBC	66	C10orf10	ANP32E	HDLBP
17	SCG5	RIN2	CANX	67	HSPA8	TBL1XR1	HNRNPK
18	ALDH1A1	GNAS	PAM	68	REG1B	GNB2L1	SCARB2
19	TM4SF4	SCG5	RPS6	69	P4HB	SLC22A17	RPL13A
20	REG3A	CSNK1A1	SERPINA3	70	LCN2	PAFAH1B2	LINC00657
21	GAPDH	PTEN	EIF4G2	71	PKM	RTN4	DSP
22	PPP1CB	TSPYL1	RPS4X	72	ATP6V0B	TMED4	SPINT2
23	ACTB	C6orf62	HSPA5	73	PSAP	CST3	REG1B
24	PRSS1	RPL3	ITGB1	74	LRRC75A-AS1	CD63	HNRNPC
25	RBP4	DPYSL2	IAPP	75	S100A11	TOB1	RPL15
26	GDF15	UBC	TPT1	76	MUC13	HLA-A	ENO1
27	COX8A	SCG2	RPL5	77	MAP1B	CLU	RPS11
28	ALDOA	ALDH1A1	SLC7A2	78	CD59	TTC3	GANAB
29	PDK4	PFKFB2	HNRNPA1	79	SLC30A8	RPS11	CDH1
30	RPL8	CPE	ANXA2	80	CPE	G6PC2	PEG10
31	H3F3B	C10orf10	RPL7	81	CLPS	GRN	CLDN4
32	IGFBP7	TMBIM6	RPS18	82	CTSD	SERPINA1	GSTP1
33	S100A6	CRYBA2	PCSK1	83	ATP1B1	SSR4	TUBA1A
34	EEF2	FTX	ATP1A1	84	OLFM4	RPS6	RPS27A
35	TIMP1	HSPA8	IDS	85	TAGLN2	OAZ1	PRPF8
36	CFL1	HSP90AA1	GDF15	86	SCGN	MARCKS	HSPB1
37	GRN	H3F3B	RPS3	87	SERPING1	RPL15	RPS8
38	SPINT2	SLC30A8	RPSA	88	WFS1	SQSTM1	RPS12
39	SQSTM1	TLK1	CSDE1	89	LAPTM4A	RASD1	ACLY
40	KRT19	ETNK1	CLTC	90	TAAR5	DSP	MSN
41	CD63	B2M	RPL10	91	SLC22A17	COX8A	HNRNPA2B1
42	SLC40A1	DDX5	YWHAZ	92	RPL3	TIMP1	CTNNB1
43	G6PC2	FOS	RPL3	93	HERPUD1	ATP1B1	MORF4L1
44	REG1A	MAFB	SLC30A8	94	CD24	WFS1	SERINC1
45	DDX5	CD59	RPL6	95	CALR	PRDX3	KRT19
46	PCBP1	TM4SF4	TMSB10	96	CLDN7	CHP1	NCL
47	C6orf62	TMEM33	CD44	97	LAMP2	YWHAE	GPX4
48	CRYBA2	CAPZA1	NPM1	98	CST3	FAM46A	GNB1
49	CD74	CALM2	B2M	99	TMBIM6	RUFY3	RPS7
50	HLA-E	GPX3	PABPC1	100	CTSB	C4orf48	SEP2
	alpha	beta	delta		gamma	acinar	ductal

Supplementary Table 6: List of top 100 high weighted genes from the mouse kidney, step 1 of tree-based recursive deconvolution.

Rank	Beckerman	Craciun	Arvaniti	Rank	Beckerman	Craciun	Arvaniti
1	Kap	Malat1	Malat1	51	Cycs	Dbi	Rps14
2	mt-Atp6	Kap	Kap	52	Rplp1	Rps18	Cox4i1
3	Gpx3	mt-Atp6	Gpx3	53	Rpl23	Rps14	Rpl26
4	mt-Co1	Gpx3	S100g	54	Gatm	Cycs	Cox5a
5	mt-Cytb	mt-Co1	Ftl1	55	Rpl32	Cox4i1	Rps19
6	S100g	mt-Cytb	Fth1	56	Cyb5a	Uqcrb	Rpl10
7	mt-Co3	S100g	Rps29	57	Acsm2	Ndrg1	Ttc36
8	mt-Co2	mt-Co3	Xist	58	Guca2b	Rpl10	Rpl35
9	mt-Nd4	mt-Co2	Rpl37a	59	Uqcrb	Rpl26	Gm8730
10	mt-Nd1	mt-Nd4	Rpl41	60	Rps14	Rps19	Dnase1
11	Ftl1	mt-Nd1	Fxyd2	61	Cox4i1	Acsm2	Itm2b
12	Fth1	Ftl1	Rpl38	62	Rpl26	Rpl35	Rpl35a
13	Rps29	Fth1	Rpl37	63	Cox5a	Cyb5a	Rps24
14	mt-Nd2	Rps29	Miox	64	Rps19	Miox	Gm10260
15	mt-Nd3	mt-Nd2	Eef1a1	65	Ttc36	Itm2b	Atp5l
16	Rpl37a	mt-Nd4l	Rpl39	66	Rpl10	Rpl35a	Slc34a1
17	Rpl41	mt-Nd3	Cox6c	67	Dnase1	Atp5l	Aldob
18	Fxyd2	Rpl37a	Rps28	68	Rpl35	Gm8730	Cela1
19	Rpl38	Rpl41	Rps27	69	Rpl35a	Akr1c21	Ass1
20	Rpl37	Xist	Cndp2	70	Atp5l	Rpl28	Prdx1
21	Miox	Fxyd2	Cyp4b1	71	Rps24	Slc34a1	Rpl28
22	Eef1a1	Rpl37	Ndufa4	72	Slc34a1	Prdx1	Rpl23a
23	Rpl39	Rpl38	Akr1c21	73	Gm8730	Aldob	Rpl6
24	Cox6c	Eef1a1	Atp1a1	74	Itm2b	Rps27a	Pck1
25	Rps28	Spink1	Acy3	75	Aldob	Cox6a1	Gm10709
26	mt-Nd5	Rpl39	Atp5k	76	Cela1	Rps24	2010107E04Rik
27	Rps27	Rps28	Cox7c	77	Ass1	Rpl23a	Cox6a1
28	Cndp2	Cox6c	Klk1	78	Prdx1	Rps4x	Slc25a5
29	Cyp4b1	Rps27	Ubb	79	Rpl28	Gm10709	Rps4x
30	Ndufa4	mt-Nd5	Atp5e	80	Rpl6	Slc25a5	Rps27a
31	Akr1c21	mt-Atp8	Rps2	81	Rpl23a	Ppia	Ldhb
32	Atp1a1	Atp1a1	Ndrg1	82	Pck1	Cox5a	Cox6b1
33	Acy3	Cox7c	Rps23	83	2010107E04Rik	Rpl13	Rpl18a
34	Atp5k	Ubb	Gm10076	84	Cox6a1	Cox6b1	Calb1
35	Cox7c	Atp5e	Prdx5	85	Gm10709	Cox7a2	Rpl13
36	Klk1	Atp5k	Rps18	86	Slc25a5	Gatm	Atp5b
37	Atp5e	Ndufa4	Tpt1	87	Rps27a	Ass1	Rpl13a
38	Ubb	Rps2	Chchd10	88	Rps4x	Ndufa3	Cox7a2
39	Rps2	Rps23	Rplp0	89	Ldhb	Rpl18a	Ndufa3
40	Ndrg1	Gm10076	Dbi	90	Cox6b1	Cyp4b1	Slc27a2
41	Rps23	Klk1	Rpl29	91	Calb1	Atp5j	Actb
42	Gm10076	Rps21	Rps21	92	Atp5b	Cox8a	Ppia
43	Prdx5	Rpl29	Rplp1	93	Cox7a2	Acy3	Rpl36a
44	Chchd10	Prdx5	Cycs	94	Rpl18a	Rpl36a	Atp5j
45	Tpt1	Rplp1	Rpl23	95	Ndufa3	Actb	Chpt1
46	Rps18	Tpt1	Rpl32	96	Slc27a2	Ndufa13	Rps15a
47	Dbi	Rpl23	Gatm	97	Rpl13	Rpl13a	Hrsp12
48	Rps21	Rpl32	Acsm2	98	Rpl36a	Ttc36	Ndufa13
49	Rplp0	Chchd10	Guca2b	99	Ppia	2010107E04Rik	Cox8a
50	Rpl29	Rplp0	Uqcrb	100	Atp5j	Gm10260	Ugt2b38
	PT	DCT	CD-IC		Podo	T lymph	

Supplementary Table 7: List of top 100 high weighted genes from the mouse kidney, step 2 of tree-based recursive deconvolution.

**Immune**							
Rank	Beckerman	Craciun	Arvaniti	Rank	Beckerman	Craciun	Arvaniti
1	Cd74	Apoe	Cd74	26	C1qb	Npc2	C1qb
2	Lyz2	S100a6	Lyz2	27	Nkg7	Gzma	Nkg7
3	Ccl5	S100a4	Ccl5	28	Ccl4	Capza2	Vim
4	H2-Aa	Psap	H2-Aa	29	Vim	Ly6e	Ccl4
5	H2-Ab1	Nkg7	H2-Ab1	30	Ly6c2	Ly6c2	Ly6c2
6	Tmsb10	Crip1	Tmsb10	31	Ms4a4b	Serinc3	Ms4a4b
7	Gzma	Cd3g	Gzma	32	Sat1	Fos	Sat1
8	H2-Eb1	Ccl3	H2-Eb1	33	C1qc	Pou2f2	C1qc
9	Plac8	Ccnd2	Plac8	34	S100a10	Ctsz	S100a10
10	Cst3	Slpi	Cst3	35	H3f3a	Cd74	H3f3a
11	Ifi27l2a	Gm2a	Ifi27l2a	36	Ctss	Il7r	Ctss
12	Slpi	Ssr4	Slpi	37	Gngt2	H2afy	Gngt2
13	Ifitm3	Lck	Ifitm3	38	S100a6	Ctsb	S100a6
14	Apoe	Spi1	Apoe	39	S100a4	Ifngr1	S100a4
15	Tyrobp	Fxyd5	Tyrobp	40	Lst1	Tgfb1	Lst1
16	Actg1	Ccl4	Actg1	41	Klf2	Sub1	Klf2
17	Crip1	Gzmb	Crip1	42	Msrb1	Socs2	Msrb1
18	Fcer1g	Cnn2	Fcer1g	43	H2afz	Ifitm3	H2afz
19	Cebpb	Id2	Cebpb	44	Wfdc17	Itgb7	Wfdc17
20	C1qa	Cybb	C1qa	45	Arpc1b	Cd79a	Arpc1b
21	AW112010	Sep1	AW112010	46	Ifitm2	Ltb	Ltb
22	Ly6e	Hsp90b1	Ly6e	47	Ltb	Fyb	Ifitm2
23	Id2	Itgb2	Id2	48	S100a11	Tspan32	S100a11
24	Psap	Ccl6	Psap	49	Lgals3	Sat1	Mzb1
25	Lgals1	Lsp1	Lgals1	50	Mzb1	Xbp1	Lgals3
**Epithelial**							
Rank	Beckerman	Craciun	Arvaniti	Rank	Beckerman	Craciun	Arvaniti
1	Hbb-bs	Hbb-bs	Hbb-bs	26	Gm5424	Slc12a3	Slc22a28
2	Hba-a1	Hba-a1	Hba-a1	27	Slc12a3	Slc22a28	Slc22a29
3	Umod	Slco1a1	Slco1a1	28	Nrp1	Slc22a29	Emcn
4	Slco1a1	Slc22a6	Slc22a6	29	Igfbp5	Ly6c1	Car12
5	Slc22a6	Pvalb	Nat8	30	Ehd3	Car12	Aspdh
6	Pvalb	Nat8	Pvalb	31	Slc22a28	Aspdh	Akr1c14
7	Nat8	Umod	Mep1a	32	Slc12a1	Igfbp5	Ly6c1
8	Mep1a	Mep1a	Umod	33	Slc22a29	Akr1c14	Hexb
9	Egf	Slco1a6	Slco1a6	34	Car12	Atp6v1g3	BC035947
10	Slco1a6	Ces1f	Ces1f	35	Aspdh	Ehd3	Igfbp5
11	Ces1f	Hbb-bt	Hbb-bt	36	Akr1c14	Hexb	Atp6v1g3
12	Hbb-bt	Egf	Snhg11	37	Kdr	Slc12a1	Nrp1
13	Snhg11	Snhg11	Tmigd1	38	Atp6v1g3	BC035947	Slc13a1
14	Tmigd1	Tmigd1	Egf	39	Hsd11b2	Slc13a1	Slc12a1
15	Acsm3	Acsm3	Acsm3	40	Hexb	Col6a6	Col6a6
16	Slc22a30	Slc22a30	Slc22a30	41	Eng	Gm4450	Gm4450
17	Gm11128	Cyp2a4	Gm11128	42	BC035947	Kdr	Adamts15
18	Aqp2	Hba-a2	Cyp2a4	43	Pi16	Adamts15	Ehd3
19	Cyp2a4	Aqp2	Hba-a2	44	Slc13a1	Hsd11b2	Aspa
20	Fxyd4	Aqp1	Gm5424	45	Col6a6	Aspa	Mogat1
21	Emcn	Gm5424	Slc17a1	46	Gm4450	Apela	D630029K05Rik
22	Aqp1	Slc17a1	Aqp1	47	Egfl7	Mogat1	Gm15638
23	Hba-a2	Plpp1	Aqp2	48	Adamts15	D630029K05Rik	Hsd11b2
24	Ly6c1	Fxyd4	Slc12a3	49	Meis2	Eng	Akr1c18
25	Slc17a1	Emcn	Fxyd4	50	Aspa	Gm15638	Smlr1
	PT	DCT	CD-IC	LOH	CD-PC	Endo	Podo
	Neutro	T lymph	Macro	Fib	B lymph	NK	

Supplementary Table 8: Starting points for convergence analysis

Cell type	EQ	SP1	SP2	SP3	SP4	SP5	SP6	SP7	SP8
alpha	0.25	0.4	0.2	0.2	0.2	0.7	0.1	0.1	0.1
beta	0.25	0.2	0.4	0.2	0.2	0.1	0.7	0.1	0.1
delta	0.25	0.2	0.2	0.4	0.2	0.1	0.1	0.7	0.1
gamma	0.25	0.2	0.2	0.2	0.4	0.1	0.1	0.1	0.7

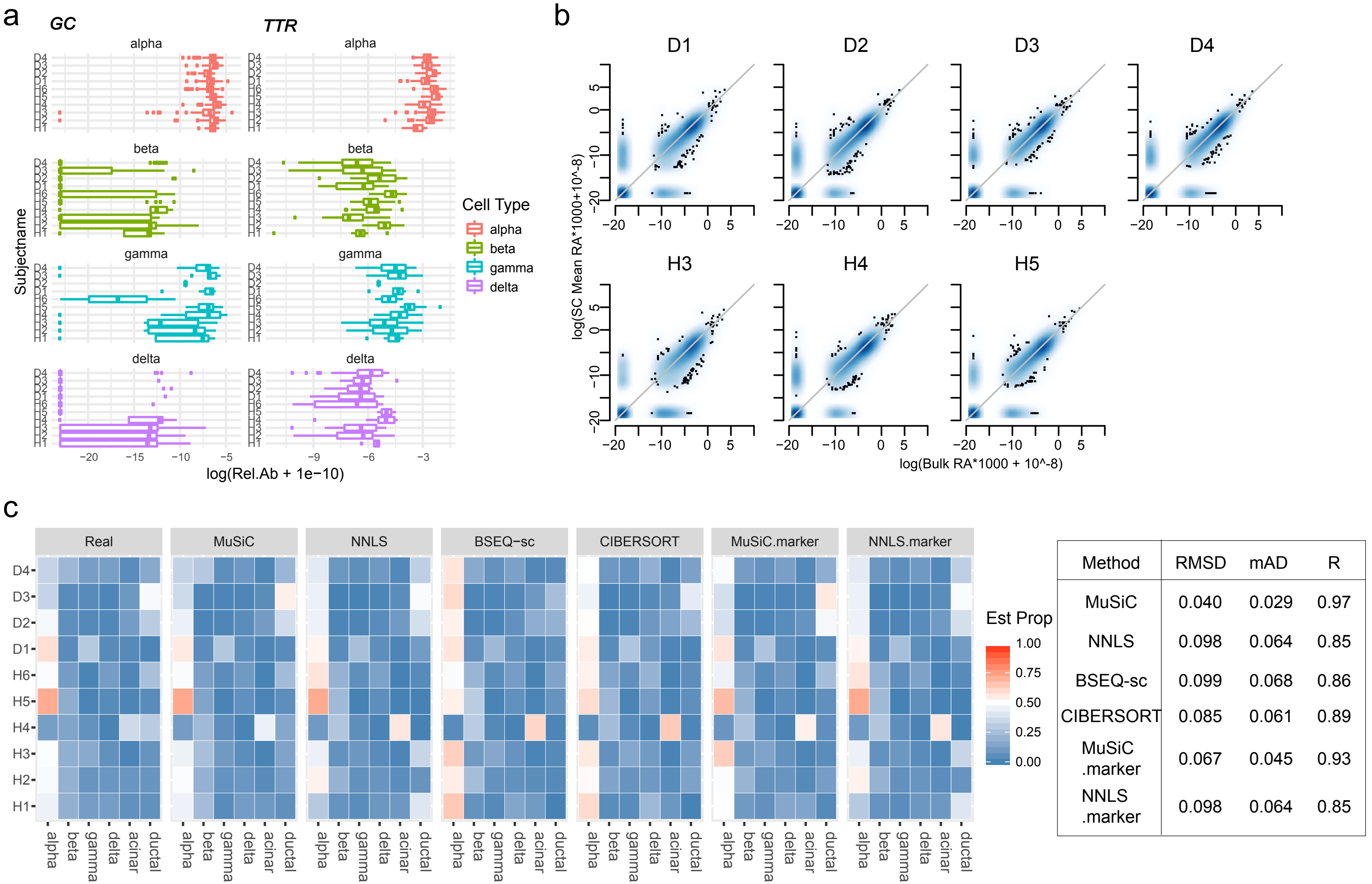

Method	RMSD	mAD	R
MuSiC	0.040	0.029	0.97
NNLS	0.098	0.064	0.85
BSEQ-sc	0.099	0.068	0.86
CIBERSORT	0.085	0.061	0.89
MuSiC .marker	0.067	0.045	0.93
NNLS .marker	0.098	0.064	0.85

Supplementary Figure 1: Exploratory analysis of single-cell RNA-seq data from Segerstolpe et al.
a. Example of cross-subject and cross-cell variation in cell type specific gene expression. The boxplot contains 4 cell types: alpha, beta, gamma, and delta cells from Segerstolpe et al. single-cell RNA-seq data. The x-axis is the log transformed average relative abundance across cells from the same cell type, and the y-axis is the subject label. The relative abundance of gene *GC* is widely spread across the x-axis while the relative abundance of gene *TTR* is more concentrated across subjects. We consider gene *GC* as non-informative and *TTR* as informative. **b**. Comparison of log transformed relative abundance levels between real bulk tissue RNA-seq data and artificially constructed bulk RNA-seq data for the same subject. Single-cell and bulk tissue RNA-seq data are both from Segerstolpe et al. Each dot represents a gene and the gray line is x=y. **c**. Heatmap of true and estimated cell type propo-rtions. In addition to the four methods described in the main text, we also evaluated the estimates given by MuSiC and NNLS when using only the marker genes used in BSEQ-sc. Source data are provided as a Source Data file.

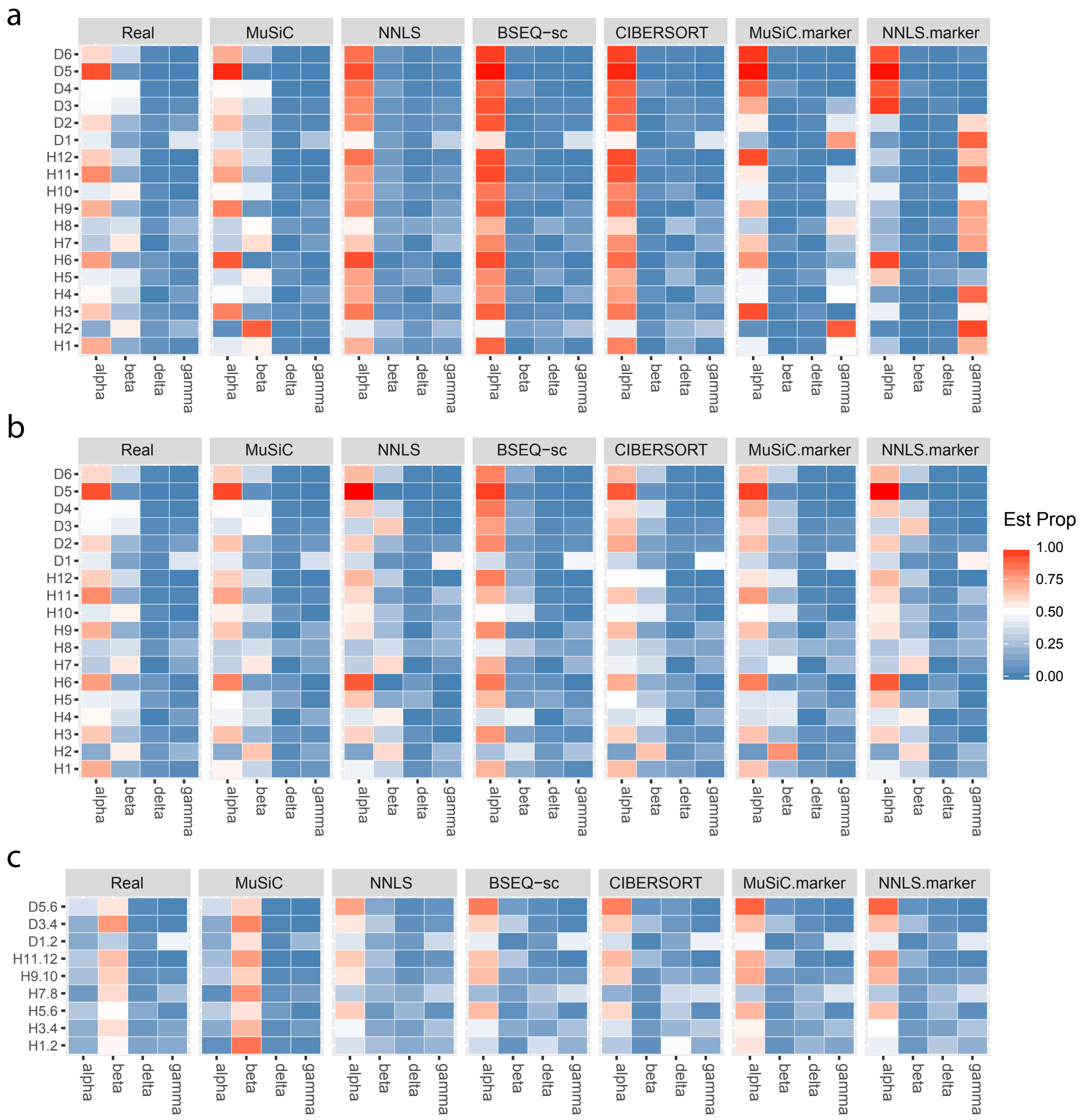

Segerstolpe et al. as reference

Method	RMSD	mAD	R
MuSiC	0.10	0.06	0.94
NNLS	0.17	0.12	0.81
BSEQ-sc	0.22	0.15	0.79
CIBERSORT	0.21	0.15	0.76
MuSiC .marker	0.25	0.18	0.60
NNLS .marker	0.34	0.26	0.30

Xin et al. as reference

Method	RMSD	mAD	R
MuSiC	0.05	0.033	0.98
NNLS	0.10	0.072	0.92
BSEQ-sc	0.13	0.088	0.87
CIBERSORT	0.07	0.053	0.95
MuSiC .marker	0.07	0.048	0.96
NNLS .marker	0.10	0.072	0.92

Method	RMSD	mAD	R
MuSiC	0.10	0.07	0.94
NNLS	0.25	0.19	0.23
BSEQ-sc	0.28	0.21	0.16
CIBERSORT	0.30	0.24	0.03
MuSiC .marker	0.31	0.23	0.10
NNLS .marker	0.31	0.23	0.08

Supplementary Figure 2: Heatmaps of true and estimated cell type proportions of artificial bulk data constructed using single-cell RNA-seq data from Xin et al.

a. Deconvolution results when the single-cell reference is from the 6 healthy subjects of Segerstolpe et al. with leave-one-out, i.e., for each subject under deconvolution, only single-cell data from the remaining 5 subjects were used as single-cell reference.

b. Deconvolution results when the single-cell reference is from the 12 healthy subjects of Xin et al. with leave-one-out, i.e., for each subject under deconvolution, only single-cell data from the remaining 11 subjects were used as single-cell reference. **c**.The cell type proportions for the artificial bulk data are manually adjusted so that beta cells are the dominant cell type, as expected in real bulk tissue. Alpha cells dominate in the scRNA-seq data due to dissociation and capture bias. Thus, this analysis mirrors the real data analysis scenario where cell type proportions differ substantially between scRNA-seq reference and bulk tissue. In more detail, we combined cells from two subjects as one artificial bulk tissue RNA-seq dataset, for example, H1.2 combined cells from subject H1 and H2. Then we dropped 75% of the alpha cells at random. The single-cell reference is from the 6 healthy subjects of Segerstolpe et al. Here, all methods that rely on pre-selected marker genes from CIBERSORT are heavily biased by the cell type proportions in the single cell reference, and miss the true cell type proportions in the bulk tissue data. In comparison, MuSiC is able to adjust to the difference between scRNA-seq reference and bulk data. Source data are provided as a Source Data file.

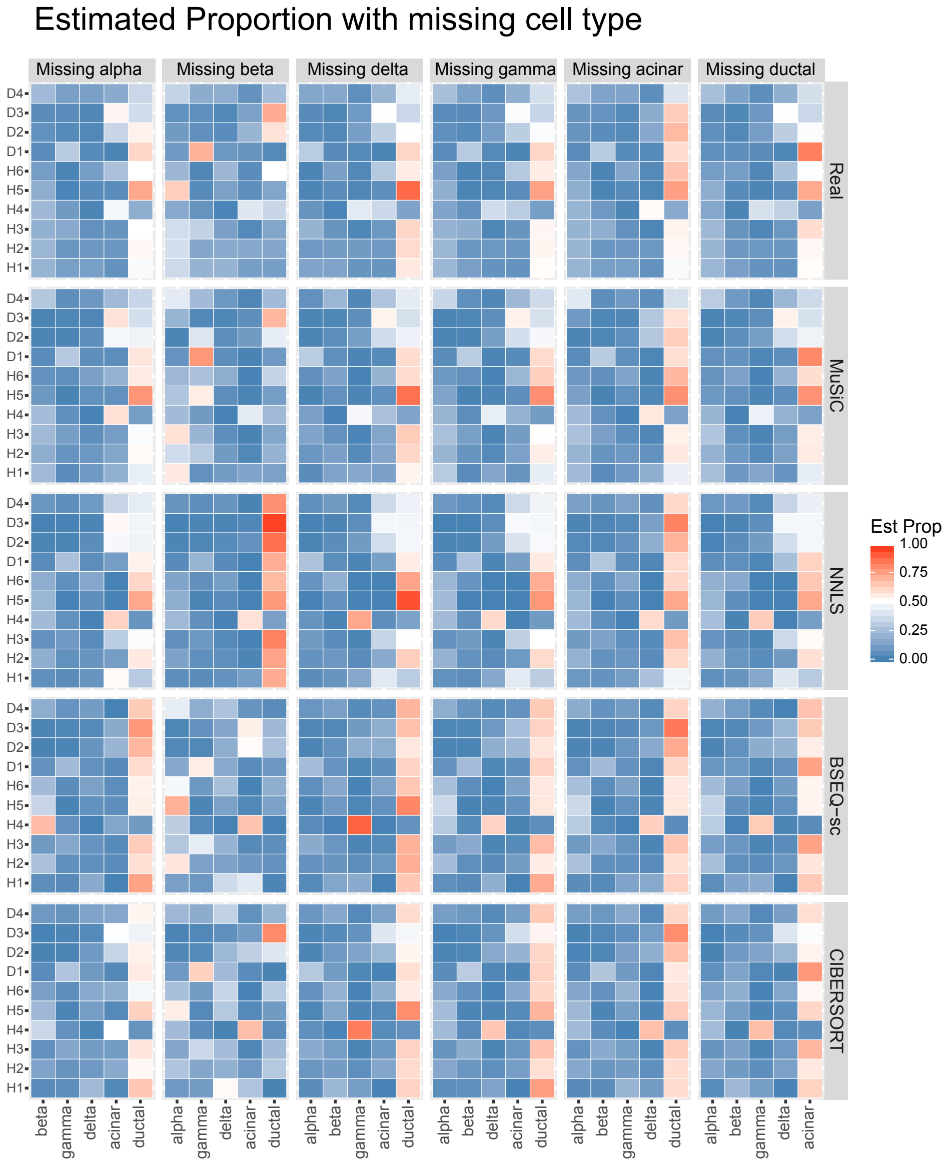

Supplementary Figure 3: Heatmaps of true and estimated cell type proportions with missing cell types in single-cell reference.

The artificial bulk data and the single-cell reference are both from Segerstolpe et al. We constrained our analysis to the 6 major cell types: alpha, beta, delta, gamma, acinar and ductal cells. The artificial bulk data is constructed by summing read counts from the 6 major cell types while the single-cell reference contains only 5 cell types (the column header shows the cell type that is missing in the single-cell reference). The x-axis labels cell types used in the single-cell reference and the y-axis shows the subject label. The top panel shows the true composition, while panels below it show the results from each method. See **Supplementary Table 3** for detailed evaluation results. Source data are provided as a Source Data file.

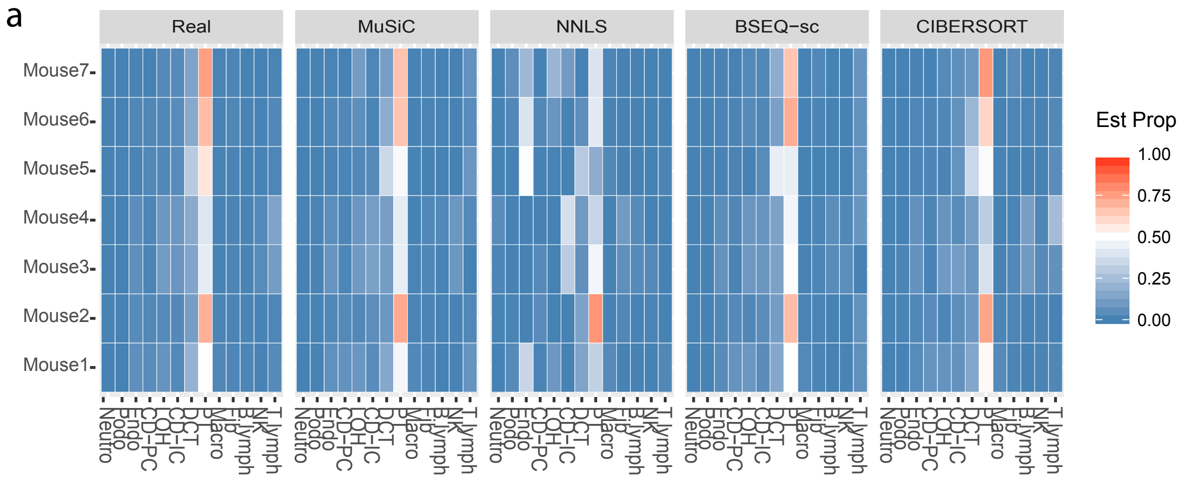

Method	MuSiC	NNLS	BSEQ-sc	CIBERSORT
RMSD	0.030	0.115	0.033	0.031
mAD	0.018	0.061	0.018	0.019
R	0.981	0.711	0.977	0.980

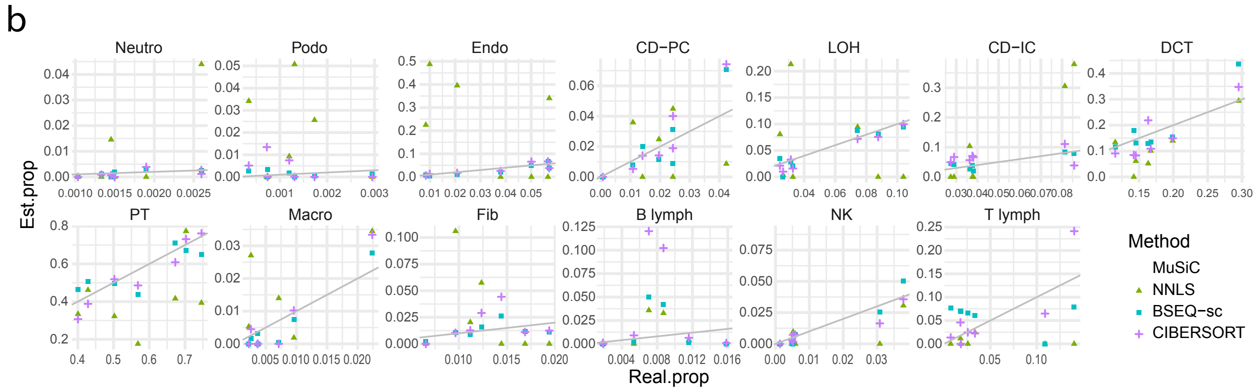

Supplementary Figure 4: Benchmark evaluation using mouse kidney single-cell RNA-seq data from Park et al.

The artificial bulk RNA-seq data is constructed by summing read counts across cells in all 16 cell types while the single-cell reference only consists of 13 cell types. The other 3 cell types were discarded in the single-cell reference because they are too rare.

a. Heatmap of estimated cell type proportions and evaluation results. **b**. Scatter plot of real cell type proportions versus estimated cell type proportions. Source data are provided as a Source Data file.

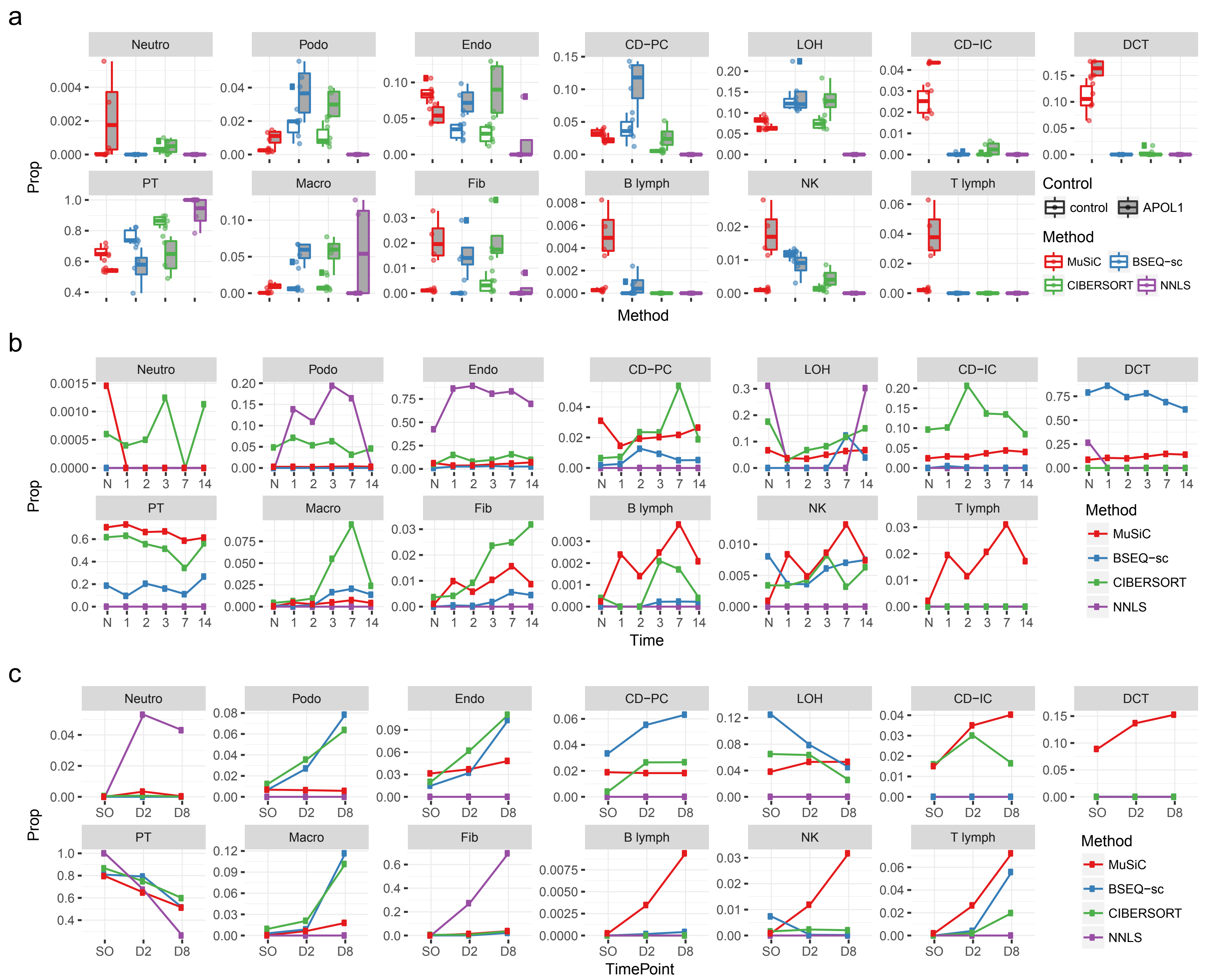

Supplementary Figure 5: Estimated cell type proportions of the 13 cell types in three real mouse bulk RNA-seq datasets. **a**. Boxplot of estimated cell type proportions of 10 mice (4 APOL1 disease mice and 6 control mice) from Beckerman et al. **b**. Line plot of cell type proportion changes after FA induction (Craciun et al.) at 6 time points. There are 3 replicates at each time point and the average proportions are plotted. N: normal. **c**. Line plot of cell type proportions of control (Sham operated mice), 2 days and 8 days after UUO (Arvaniti et al.). Source data are provided as a Source Data file.

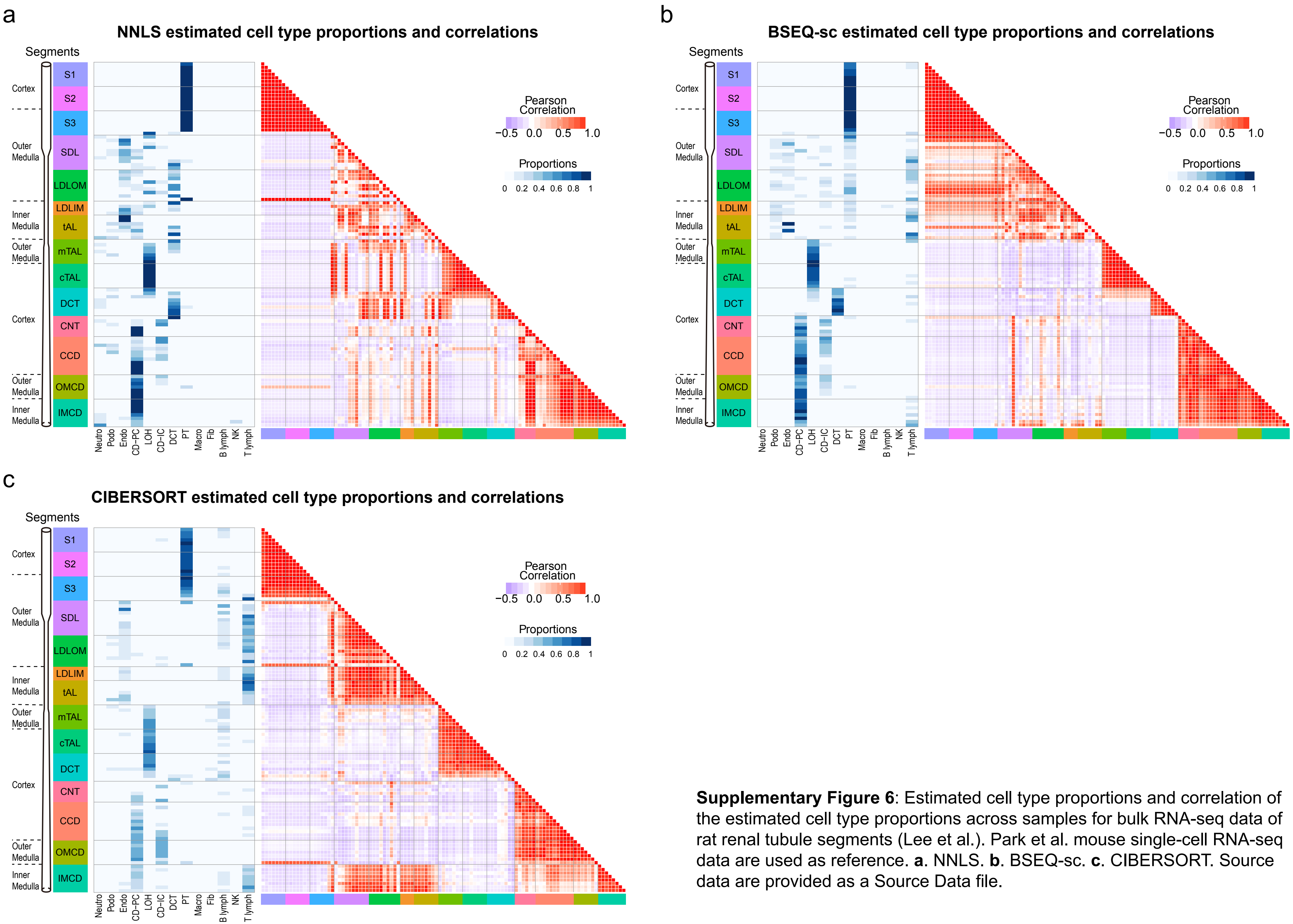

Supplementary Figure 6: Estimated cell type proportions and correlation of the estimated cell type proportions across samples for bulk RNA-seq data of rat renal tubule segments (Lee et al.). Park et al. mouse single-cell RNA-seq data are used as reference. **a**. NNLS. **b**. BSEQ-sc. **c**. CIBERSORT. Source data are provided as a Source Data file.

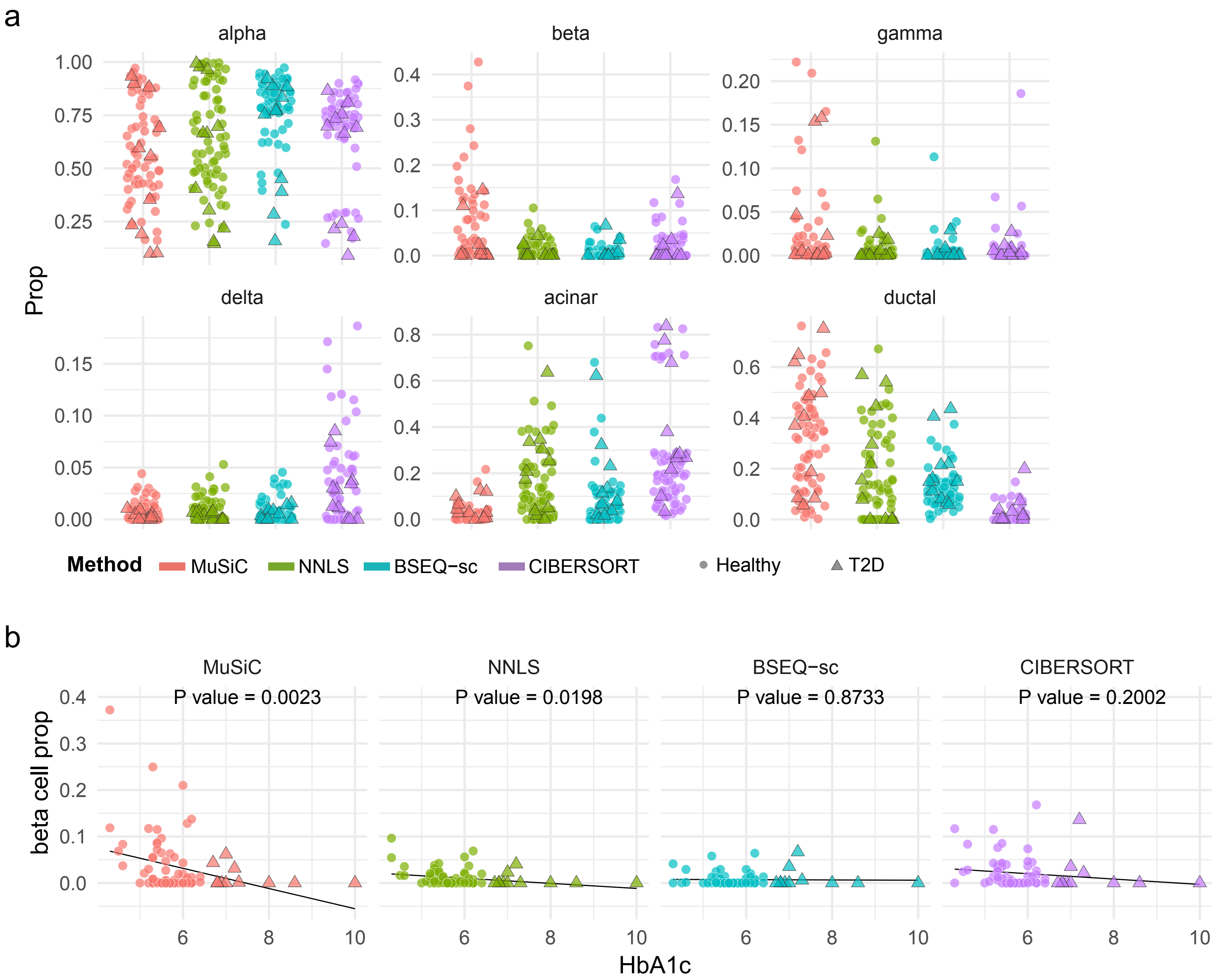

Supplementary Figure 7: Estimated cell type proportions of the pancreatic islet bulk RNA-seq data in Fadista et al. with single cell reference from Baron et al.
The analysis is similar to **Figure 2c-d** in the main text except that the single-cell reference are based on the three healthy subjects from Baron et al. and the MuSiC estimation was adjusted for protocol bias as described in the **Methods** section. **a**. Jitter plot of the estimated cell type proportions for Fadista et al. subjects, color-coded by deconvolution methods. 77 out of the 89 subjects from Fadista et al. that have recoded HbA1c levels are plotted. T2D subjects are denoted as triangles. **b**. HbA1c levels vs beta cell type proportions estimated by each of the four methods. The reported p-values are from single variable regression β cell proportions ~ HbA1c. Multivairiable regression results adjusting for age, BMI and gender are reported in **Supplementary Table 2**. Source data are provided as a Source Data file.

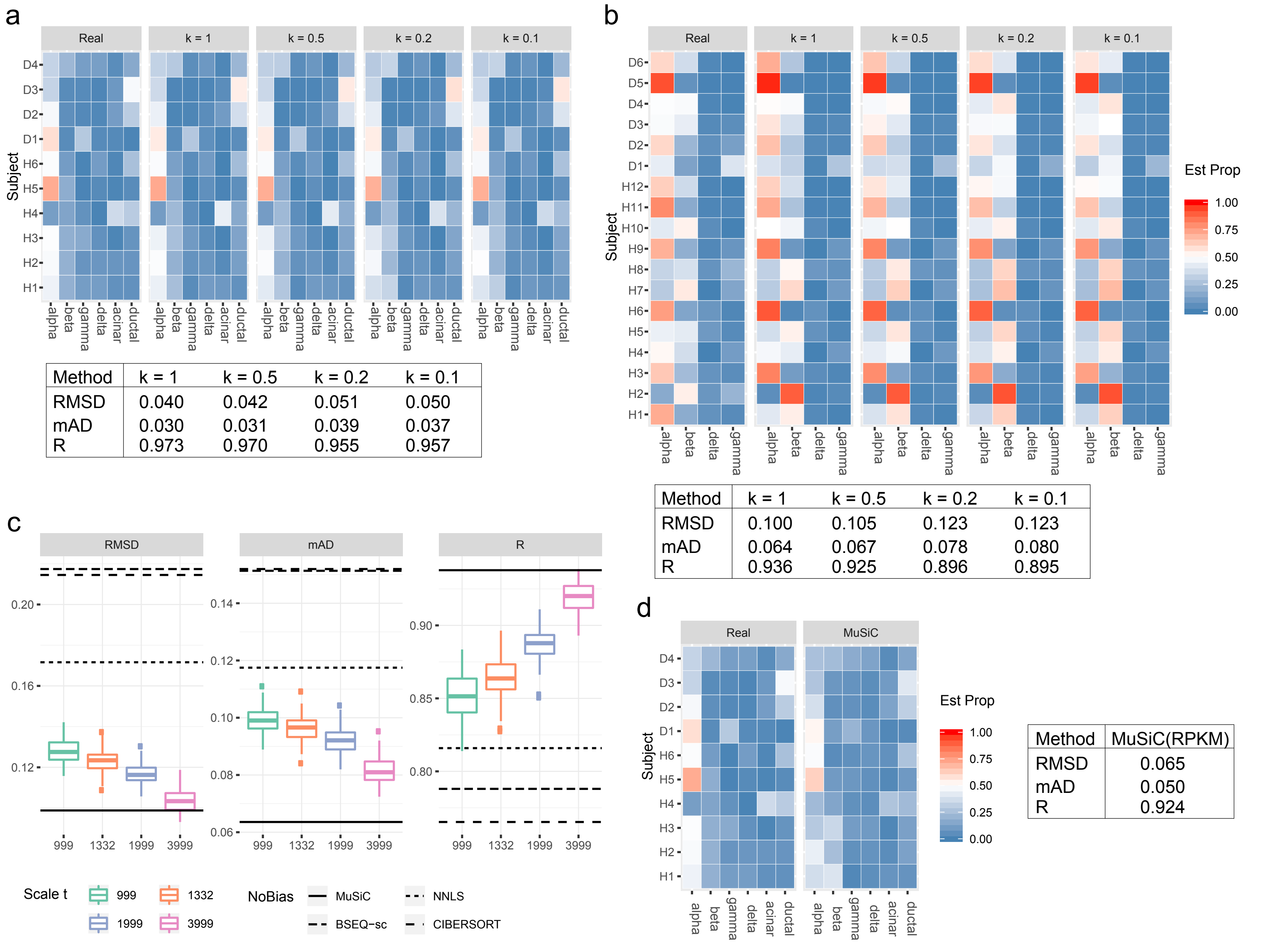

Method	k = 1	k = 0.5	k = 0.2	k = 0.1
RMSD	0.040	0.042	0.051	0.050
mAD	0.030	0.031	0.039	0.037
R	0.973	0.970	0.955	0.957

Method	k = 1	k = 0.5	k = 0.2	k = 0.1
RMSD	0.100	0.105	0.123	0.123
mAD	0.064	0.067	0.078	0.080
R	0.936	0.925	0.896	0.895

Method	MuSiC(RPKM)
RMSD	0.065
mAD	0.050
R	0.924

Supplementary Figure 8: Benchmark evaluation of robustness of MuSiC.
a. and **b.** evaluate the impact of different dropout rate in scRNA-seq (**Supplementary Note 5**). **a.** and **b.** show heatmaps of MuSiC estimated cell type proportions. The single-cell reference is based on six healthy subjects from Segerstolpe et al. with different dropout rates. The artificial bulk data of **a.** is constructed by Segerstolpe et al. while **b.** is constructed by Xin et al. **c.** Evaluation of the impact of biased relative abundance 84 in the single-cell reference (**Supplementary Note 4**). Boxplot shows three evaluation metrics from 100 simulations of MuSiC estimated cell type proportions with biased relative abundance, color-coded by scale parameter of Dirichlet distribution. The horizontal lines show the evaluation metrics of four methods without bias in the single-cell reference. **d.** Heatmap of MuSiC estimated cell type proportions with RPKM as the input. The artificial bulk data and single-cell reference are both from Segerstolpe et al. The estimation follows leave-out-one rule. We utilized the average library size ratio of the six healthy subjects from Segerstolpe et al. as the ratio of cell size. Source data are provided as a Source Data file.

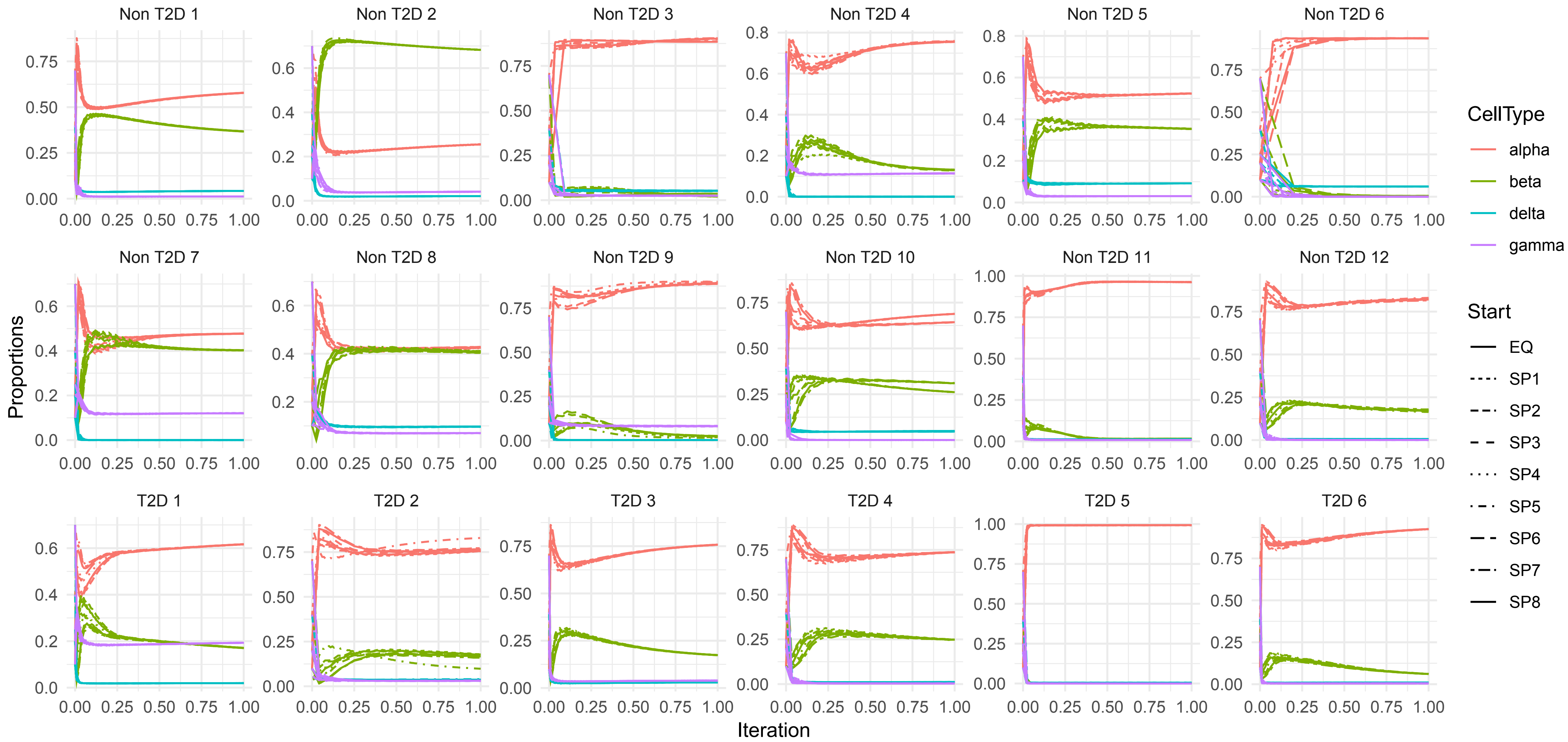

Supplementary Figure 9: Convergence of MuSiC with different starting points.
The evaluation is performed on artificial bulk data, constructed by single-cell data from Xin et al. while the single -cell reference is from Segerstolpe et al. We evaluate the convergence of MuSiC with nine different starting points of four cell types in **Supplementary Table 8**. The iteration numbers are normalized between 0 and 1 for comparison. We plotted the normalized iteration against estimated proportions for each subjects in Xin et al. colored by cell types. From different starting points, estimated cell types converged to the same proportions.

Reference

1. Xin, Y. *et al.* RNA sequencing of single human islet cells reveals type 2 diabetes genes. *Cell metabolism* **24**, 608-615 (2016).
2. Fadista, J. *et al.* Global genomic and transcriptomic analysis of human pancreatic islets reveals novel genes influencing glucose metabolism. *Proceedings of the National Academy of Sciences* **111**, 13924-13929 (2014).
3. Segerstolpe, Å. *et al.* Single-cell transcriptome profiling of human pancreatic islets in health and type 2 diabetes. *Cell metabolism* **24**, 593-607 (2016).
4. Jia, C. *et al.* Accounting for technical noise in differential expression analysis of single-cell RNA sequencing data. *Nucleic acids research* **45**, 10978-10988 (2017).
5. Baron, M. *et al.* A Single-Cell Transcriptomic Map of the Human and Mouse Pancreas Reveals Inter- and Intra-cell Population Structure. *Cell Syst* **3**, 346-360 e4 (2016).
6. Park, J. *et al.* Single-cell transcriptomics of the mouse kidney reveals potential cellular targets of kidney disease. *Science*, eaar2131 (2018).
